# The synthetic LPS binding peptide 19-2.5 interferes with clotting and prevents degradation of high molecular weight kininogen in plasma

**DOI:** 10.1038/s41598-020-64155-5

**Published:** 2020-04-28

**Authors:** Juliane Köhler, Johannes Ehler, Bernd Kreikemeyer, Rika Bajorath, Tobias Schürholz, Sonja Oehmcke-Hecht

**Affiliations:** 1 Institute of Medical Microbiology, Virology and Hygiene, Rostock University Medical Center, Rostock, Germany; 2 Department of Anesthesia and Intensive Care, Rostock University Medical Center, Rostock, Germany

**Keywords:** Immunology, Coagulation system, Peptides

## Abstract

Sepsis and septic shock are life-threatening conditions and remain an important medical problem, emphasizing the need to identify novel therapeutic approaches. Coagulation dysfunction, hypotension, disturbed microcirculation and multiorgan failure occur frequently. These severe conditions result from an overwhelming inflammatory response, induced by pathogen and damage associated molecular patterns (PAMPs and DAMPs) released into the bloodstream. In the present study, we demonstrated that the synthetic Lipopolysaccharid (LPS)-binding peptide 19-2.5 interferes with the activation of the coagulation and contact system. Moreover, binding of LPS to high molecular weight kininogen (HK), one of the major LPS carrier in blood, could be prevented by the peptide. Thus, peptide 19-2.5 might represent a promising target in the treatment of endotoxemia and sepsis, not only by its anti-inflammatory potential, but also by the anticoagulant effect, together with its ability to prevent degradation of HK.

## Introduction

Sepsis and septic shock are complications of bacterial infections that are associated with high mortality rates, despite the availability of antibiotics and improved intensive care. Complications from these life-threatening infections often arise when the initial appropriate host response to the invading pathogen is excessively amplified and progressively uncontrolled. A systemic activation of proteolytic host cascades, such as the coagulation and contact systems, together with a massive release of proinflammatory cytokines, can be deleterious for the host^1^.

The majority of sepsis complications is caused by a disproportionate immune response to bacterial products, mainly lipopolysaccharide (LPS) in case of Gram-negative bacteria. LPS is localized in the outer layer of the membrane and is exposed on the bacterial surface. LPS contributes to the integrity of the outer membrane and protects the bacterium against the action of bile salts and lipophilic antibiotics. In sepsis, LPS can be released continuously due to cell growth and division, but also due to killed bacteria after an antibiotic treatment^2^.

Free LPS is one of the most potent immune-stimulatory molecules, it can provoke a vigorous systemic inflammatory response and even minute doses may have lethal consequences for the host^3^. The response of host immune cells to LPS is initiated by its interaction with plasma proteins such as LPS-binding protein (LBP) and the specific receptor binding protein CD14 (soluble or membrane-bound), which finally leads to cell activation through the Toll-like receptor 4 (TLR4)^4^. Additionally, the plasma protein high molecular weight kininogen (HK) has been recently described to bind LPS and trigger inflammatory reactions. This might play a critical role for the LPS induced inflammatory response, as mice lacking HK, were resistant to LPS-induced mortality^5^. HK is mainly a cofactor of the human contact system, which consists of the serine proteases factor XI (FXI), factor XII (FXII), and plasma kallikrein (PK). HK circulates in a complex with either FXI or PK in plasma^6,7^. The contact system can initiate two pathways 1) coagulation via activation of FXII and FXI and 2) inflammation via the release of bradykinin from HK by PK. In humans, endotoxemia^8^ and sepsis in general^9,10^ are associated with activation of the contact system.

Moreover, in endotoxemia models in baboons, dogs, or rats, LPS causes the activation of FXII and PK, leading to the cleavage of HK and the release of bradykinin^11–13^, which potentiates inflammatory reactions further.

Thus, LPS triggers inflammation via different pathways and the toxic effect of LPS might be prevented by its neutralization. Recently a completely new class of peptides— synthetic anti-LPS peptides (SALPs) has been designed to neutralize LPS^14^. One of these peptides - Pep19-2.5 - was shown to be highly efficient in the neutralization of LPS and blockage of its immunopathological consequences *in vitro* and *in vivo*^14,15^. The aim of the present study was to test whether Pep19-2.5 interferes with the coagulation and contact system. We found that the peptide functions as mild anticoagulant in human plasma by inhibiting FXI. Moreover, the peptide prevented binding of LPS to HK, thereby blocking cleavage of HK, reducing procoagulant activity of PBMCs and the release of IL-6 and IL-10.

## Results

### Pep19-2.5 prolongs the intrinsic and extrinsic pathway of coagulation

**Figure 1:**
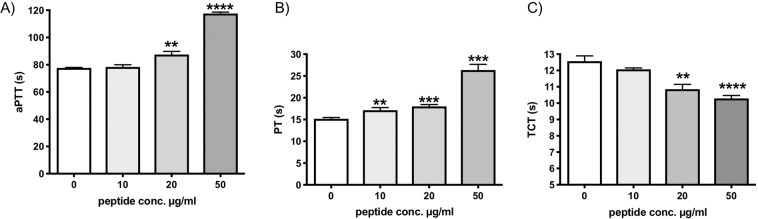
Pep19-2.5 prolongs aPTT and PT but shortens TCT. The peptide (10 - 50 µg/ml) or water (0 µg/ml) were incubated in pooled normal plasma and aPTT (**A**), PT (**B**) or TCT (**C**) were determined in a coagulometer. *p < 0.05 **p < 0.005, ***p < 0.0005, ****p < 0.0001.

Due to its amino acid sequence Pep19-2.5 is positively charged and binds LPS with high affinity^14^. We showed recently that a positively charged peptide derived from high molecular weight kininogen inhibits the intrinsic pathway of coagulation^16^, and we were interested to test Pep19-2.5 regarding a possible influence on the coagulation system. The activated partial thromboplastin time (aPTT) is the commonly used diagnostic coagulation test to measure activity of intrinsic coagulation. Incubation with increasing concentrations of the peptide in human plasma prolonged the aPTT significantly at concentrations of 20 µg/ml (Fig. 1A). Also, the prothrombin time (PT), the coagulation test for the extrinsic coagulation pathway was significantly prolonged with increasing concentrations of Pep19-2.5 (Fig. 1B). As depicted in Fig. 1A and B the effect on coagulation was dose dependent.

In contrast the thrombin clotting time (TCT), measuring thrombin-induced fibrin-network formation, was slightly but significantly shortened after incubation with Pep19-2.5, and also this effect was dose dependent (Fig. 1C).

### FXI activity is inhibited after incubation of Pep19-2.5 in plasma

**Figure 2:**
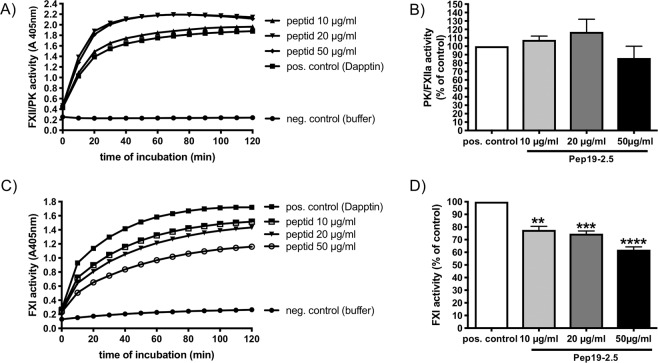
Pep19-2.5 inhibits FXI but not plasma kallikrein. Pooled diluted plasma was incubated with Pep19-2.5, and Dapptin was added for FXII activation. In the pos. control Dapptin (without peptid) and in the neg. control water was added. (**A**) PK/FXIIa activity was determined with the substrate S-2302 over a period of 120 min. One representative experiment from 2 is shown. (**B**) PK/FXIIa activity in plasma after 30 min incubation with Dapptin and different concentration of Pep19-2.5 is shown. The pos. control (Dapptin without peptide) was set to 100%. Data from 2 independent experiments were pooled. (**C**) FXI activity was determined with the substrate S-2366 over a period of 120 min. One representative experiment from 3 is shown. (**D**) FXI activity in plasma after 30 min incubation with Dapptin and different concentration of Pep19-2.5 is shown. The pos. control (Dapptin without peptide) was set to 100%. Data from 3 independent experiments were pooled. **p = 0.0014, ***p = 0.0003, ****p < 0.0001.

As the peptide prolonged the aPTT we next investigated whether it may interfere with the activation of PK or FXI in plasma. For these experiments the peptide was preincubated in diluted plasma for 1 min before the contact activator Dapptin was added. PK or FXI activity was determined using a specific chromogenic substrate for PK (S-2302) or FXI (S-2366). Incubation of Pep19-2.5 in plasma did not influence PK activity significantly, even at higher concentrations (Fig. 2A,B). However, activity of FXI was significantly inhibited and the effect was strengthened with higher peptide concentrations (Fig. 2C,D).

### Pep19-2.5 prevents degradation of HK in plasma

**Figure 3:**
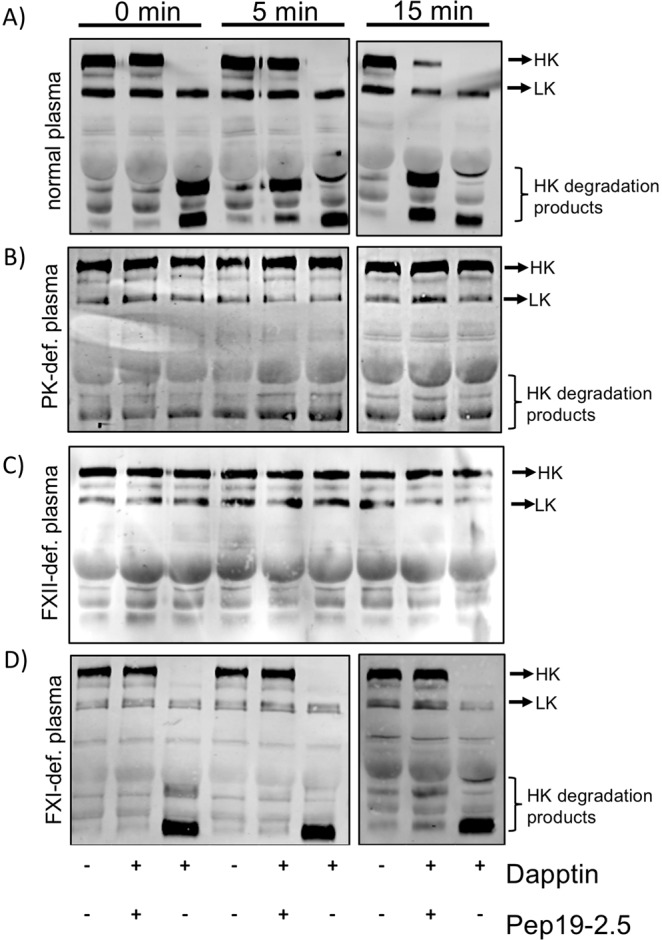
Pep19-2.5 prevents HK cleavage. Normal plasma (**A**), FXII-def. plasma (**B**), PKK def. plasma (**C**) or FXI deficient plasma were incubated with water, Dapptin or a combination of Dapptin and Pep19-2.5 (10 µg/ml) at 37 °C. After 0, 5- and 15-min samples were taken and analyzed by Western blotting with antibodies identifying HK and LK.

HK is a cofactor of the contact system and is associated with PK or FXI in plasma^6,7^. Degradation of HK occurs mainly via activated PK, but activated FXI can also cleave HK^17^. To test whether degradation of HK might be prevented by Pep19-2.5 Western Blot analysis was performed. Pooled normal plasma was incubated with Pep19-2.5 (final concentration 10 µg/ml) for 5 min, mixed with Dapptin and incubated for another 15 minutes at 37 °C. Samples were collected after 0, 5 and 15 minutes. Plasma alone or plasma treated with Dapptin only served as negative and positive controls, respectively. Western blots of the samples were stained with antibodies directed against HK, which also detect low-molecular weight kininogen (LK). LK is a shorter splice variant of HK, thus the polyclonal antiserum against HK also react with LK. Figure 3A depicts intact HK at 120 kDa and processed HK after Dapptin treatment, which triggers the conversion of HK from a single chain to a two-chain protein immediately (Fig. 3A, 0 min). When plasma was incubated with Dapptin in the presence of Pep19-2.5, cleavage of HK was blocked and intact HK could be detected, even after 15 min of incubation (Fig. 3A). In human congenital PKK or FXII-deficient plasma cleavage of HK could not be initiated by Dapptin (Fig. 3B,C), supporting the main function of FXII and PK for HK degradation. When human congenital FXI-deficient plasma was used, addition of Pep19-2.5 prevented HK degradation nearly complete over 15 min (Fig. 3D). As PK activity was not inhibited by the peptide (see above) we assume that Pep19-2.5 binds to HK, therefore interfering with its cleavage.

### Binding of Pep19-2.5 to HK

**Figure 4:**
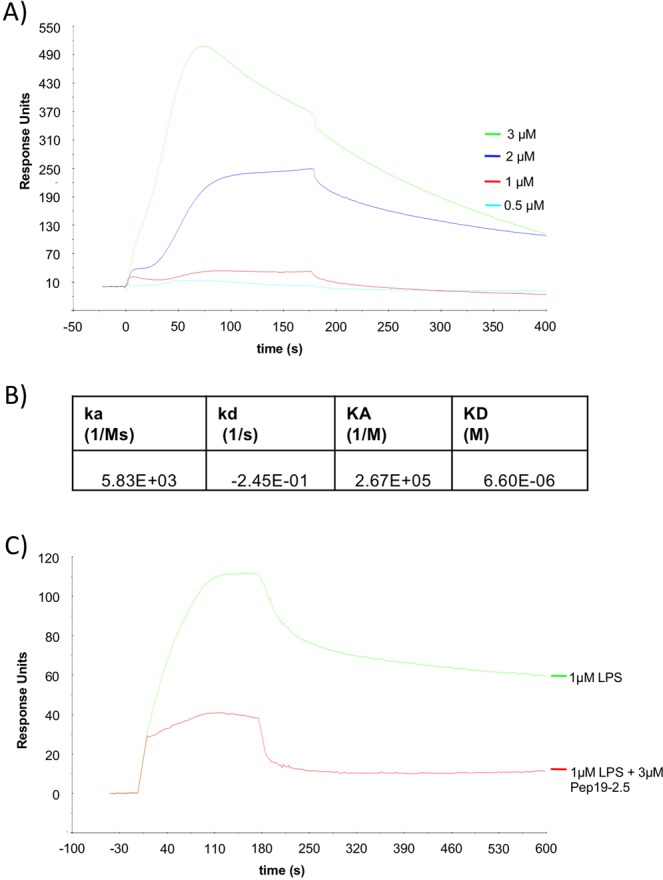
Binding properties of Pep19.2-5 measured by SPR. (**A**) HK was coupled to a sensor chip and subjected to injections with serial dilutions of Pep19.2-5 (0.5–3 µM). The kinetic and affinity parameters of this interaction are listed in (**B**). (**C**) HK was coupled to a sensor chip over which LPS (1 µM, green line) was injected, or LPS that was preincubated with 3 µM Pep19.2-5 (red line).

As the peptide prevented HK cleavage we tested whether it binds to HK by surface plasmon resonance (SPR). Sensor chips were coated with HK and probed with increasing concentrations of Pep19-2.5 (Fig. 4A). Determination of the association constants revealed that the peptide bound to HK, although with lower affinity. The estimated KD is in the micromolar range (Fig. 4B).

### Binding of Pep19-2.5 to LPS prevents binding to HK

It has been recently shown that HK is an important LPS carrier and binding of LPS to HK triggers the inflammatory response^5^. Thus, blocking LPS binding to HK with Pep19-2.5 might be a promising strategy to prevent systemic inflammatory reactions. LPS binding to HK was confirmed by SPR (Fig. 4C), however if LPS was preincubated with the peptide in a molar ratio of 1:3, binding to HK was reduced (Fig. 4C).

### Pep19-2.5 reduces procoagulant activity in LPS stimulated PBMCs

**Figure 5:**
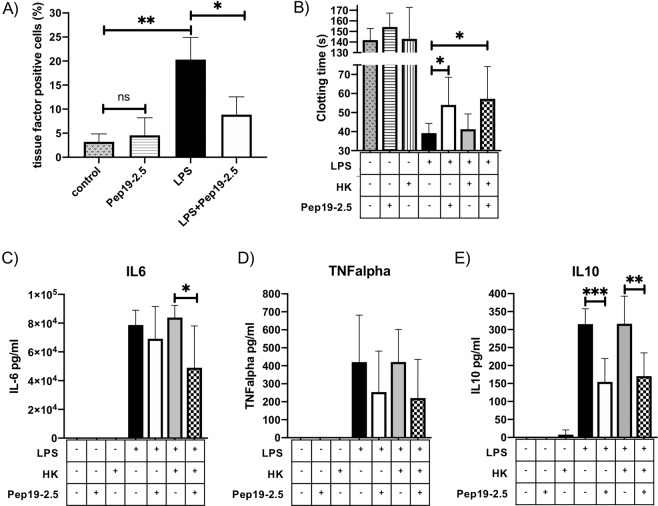
Procoagulant activity and cytokine release after treatment of PBMCs with Pep19-2.5. (**A**) Human PBMCs **(**1 × 10^6^ cells/ml) were incubated with buffer, Pep19.2-5 (100 ng/ml), LPS (10 ng/ml), or a combination of LPS and Pep19.2-5. Incubation was done on a rotator for 4 h at 37 °C. Thereafter, cells were centrifuged, and tissue factor expression of CD14 positive cells was determined by FACS analysis. **(B**) Human PBMCs **(**1 × 10^6^ cells/ml) were incubated with buffer, Pep19.2-5 (100 ng/ml), HK (1 µg/ml), LPS (10 ng/ml), or a combination of LPS and Pep19.2-5, LPS and HK, LPS and HK and Pep19.2-5. Incubation was done on a rotator for 20 h at 37 °C. Thereafter, the supernatant was kept for cytokine measurements and the cells used for clotting assays. (**B**) Cell suspensions (50 µl) were added to 50 µl of pre-warmed citrated human plasma, recalcified with 50 µl CaCl_2_ (25 mM), and the time to form a clot was measured. (**C**–**E**) Cytokine content of the supernatant was determined by ELISA. Values represent the mean ± SD of three different donors, each done in duplicates. *p < 0.05 **p < 0.005, ***p < 0.0005.

LPS triggers the induction of tissue factor in monocytes, thus we measured whether tissue factor expression can be reduced by treatment of PBMCs with the peptide. PBMCs were incubated for 4 h with LPS, Pep19-2.5 or a combination of both. LPS incubation increased tissue factor positive monocytes significantly to about 20%. Treatment with Pep19-2.5 reduced tissue factor positive monocytes significantly to less than 10% (Fig. 5A). Treatment of cells with the peptide alone had no influence on tissue factor expression (Fig. 5A).

Procoagulant activity of PBMCs can also be monitored by addition of the cells to recalcified plasma and measuring the clotting time^18,19^. To test whether Pep19-2.5 interferes with clotting of the cells, PBMCs were incubated for 20 h with LPS, Pep19-2.5 or a combination of both. LPS challenge shorten the clotting time significantly, compared to the non-treated control (Fig. 5B). If LPS was incubated together with Pep19-2.5, the clotting time was significantly prolonged (54 sec), compared to the LPS sample (39 sec). The peptide alone did not change the clotting time of the cells, compared to the control sample.

If LPS was incubated together with HK, the clotting time was unchanged (41 sec), compared to the LPS sample. However, in the presence of the peptide, the clotting time was again significantly prolonged (Fig. 5B). This supports our data in Fig. 5A showing that Pep19-2.5 is able to prevent the expression of tissue factor in LPS stimulated monocytes.

We also measured release of IL-6, TNF-alpha and IL-10 in the supernatant of the samples (Fig. 5C–E). As expected, LPS and a combination of LPS and HK induced a robust increase of all cytokines after 20 h of incubation, however the combination of LPS and HK did not increase the concentration of cytokines further. Treatment with Pep19-2.5, decreased IL-6 content significantly in samples with LPS and HK (Fig. 5C). TNF alpha concentration varied considerably between the samples, and the decrease after treatment with the peptide was not significant (Fig. 5D). In contrast, IL-10 concentration significantly decreased after the treatment with Pep19-2.5 (Fig. 5E). Taken together, a treatment of PBMCs with Pep19-2.5 reduced the release of proinflammatory IL-6 and anti-inflammatory IL-10, even after a long period of 20 h of incubation.

## Discussion

Sepsis and septic shock are a major clinical challenge, and despite intensive efforts, there are currently no new drugs launched for the treatment of patients with severe infectious diseases. Thus, there is an urgent need for novel therapies with a broader clinical indication. Here we investigated a promising candidate – the LPS binding peptide Pep.19-2.5 – regarding its ability to interfere with coagulation and contact system activation. Both systems are activated during severe sepsis, which frequently correlates with a fatal outcome of the disease^1^.

The LPS binding peptide 19-2.5 inhibits activation of the intrinsic and extrinsic coagulation pathway, revealed by clotting assays. This effect probably results from inhibition of FXI by Pep19-2.5, as FXI activity was significantly reduced with peptide concentrations of 10 µg/ml. FXI is activated by FXII, however, also thrombin and forms of thrombin contribute directly to activation of FXI in plasma^20–22^. Intriguingly, the TCT was slightly reduced after peptide incubation. Even though a reduced TCT in this range has no clinical importance, it might be interesting to investigate this phenomenon in further studies.

Activity of PK was not influenced by the peptide. Both, FXI and PK are associated with HK in plasma, which acts also as a cofactor for their activation^23^. If the contact system becomes activated, PK cleaves HK and bradykinin will be released. Surprisingly, HK cleavage was prevented if the plasma was treated with Pep19-2.5, regardless of the fact that PK itself was not inhibited by the peptide. Thus, the mechanism of cleavage inhibition by Pep19.2-5 are supposed to be different. SPR analysis revealed that the peptide binds to HK, although with a lower affinity in the micromolar range. However, binding of the peptide to HK might cover the PK cleavage site, and, in this way prevented HK cleavage by PK.

Several studies have shown that a massive activation of the contact system can trigger the generation of pathologic kinin levels and lead to a consumption of contact factors followed by impaired hemostasis^11,24^. Moreover, HK is an important LPS carrier and binding of LPS to HK triggers the inflammatory response^5^. Here we revealed by SPR analysis that Pep19-2.5 blocks LPS binding to HK, which might be a promising strategy to prevent systemic inflammatory reactions by this pathway. This assumption is supported by earlier studies already showing that acute endotoxic shock was prevented in mice by prophylactic or therapeutic treatment with Pep19-2.5. This is accompanied by a reduction of proinflammatory cytokines such as TNF-alpha and IFN-gamma^25^. Moreover, in a Salmonella infection model in rabbits the IL6 levels were significantly reduced after a combined treatment with antibiotics and Pep19-2.5^26^. *In vitro* it has been shown that the treatment of mononuclear cells with the peptide inhibits proinflammatory cytokines after a shorter period (4 – 6 h) of LPS incubation^14^. LPS incubation is additionally followed by upregulation of tissue factor in monocytes^27,28^. In the present study we show that Pep19-2.5 prevents tissue factor expression in monocytes and therefore prevents procoagulant activity of the cells. Moreover, when mononuclear cells were challenged for 20 h with LPS or a combination of LPS and HK, the treatment with Pep19-2.5 decreased IL-6 as well as IL-10 concentrations significantly. IL-10 secretion and lymphocyte exhaustion are the main features of sepsis-induced immunosuppression^29^, and high IL-10 level contributed to higher mortality of sepsis^30,31^. Continuous release of IL-10 might amplify sepsis-induced immunosuppression and thus might augment susceptibility to secondary microbial infections^29^. As previously shown, blocking IL-10 could reverse sepsis-induced immunosuppression and improved survival in a clinically relevant animal model of sepsis^32^.

Hence, dampen the exaggerated release of pro- and anti-inflammatory cytokines by Pep19-2.5 might help to keep the balance between both processes. Moreover, the anticoagulant effect of the peptide, together with its ability to prevent degradation of HK, might prevent a massive activation of the contact system and the generation of pathologic bradykinin levels, which has to be proved in further studies.

## Methods

### Human plasma

Pooled plasma obtained from healthy donors was purchased from Affinity Biologicals Inc (Canada). PKK- and FXI-deficient plasmas were purchased from George King Bio-Medical (United States).

### Pep19-2.5

The synthesis and purification of Pep19-2.5 was described previously^14^.

### Clotting assays

All clotting times were measured using an Amelung coagulometer. Activated partial thromboplastin time (aPTT) was measured by incubating Pep19-2.5 at different concentrations with plasma for 1 minute followed by the addition of equal amounts of Dapptin, containing silica, sulfatide and phospholipids (Technoclone) for 60 seconds at 37 °C. Clotting was initiated by the addition of 25 mM CaCl_2_. For the prothrombin time assay (PT), clotting was initiated by the addition of Technoplastin HIS (PT reagent, Technoclone).

For clotting of PBMCs, 50 µl plasma was incubated with 50 µl 25 mM CaCl_2,_ and 5 × 10^5^ cells in a volume of 50 µl were added. Time until clot formation was determined.

### Chromogenic substrate assay

Pooled diluted plasma (1:10 in 15 mM HEPES) was incubated with Pep19-2.5, and Dapptin was added for contact activation. In the positive control Dapptin (without peptid) and in the negative control water was added. 1 mM of the chromogenic substrate S-2302 (for PK/FXIIa activity, Chromogenix) or S-2366 (for FXI and Prot.C activity, Chromogenix) were added and the absorbance at 405 nm was measured at 37 °C over a period of 120 min. No endogenous proteolytic activity was measured in the absence of plasma.

### Electrophoresis and Western Blot analysis

Proteins in plasma were separated and blotted as described^16^, followed by incubation with anti-human HK (Affinity Biologicals, Canada). Blots were incubated with secondary fluorophore-labeled antibodies (LI-COR) and imaged using an Odyssey Imager (LI-COR).

### Surface Plasmon Resonance (SPR)

The interactions between Pep19-2.5 (as analyte) and HK (as ligand) were analyzed with a BIAcore3000 system (Biosensor, La Jolla, CA) using CM5 sensor chips at 25 °C in HBS-EP as running buffer. HK was immobilized on a flow cell surface of the chip to a density of 1002 response units (RUs) using standard amine-coupling chemistry and the software tool “Application Wizard-Surface Preparation” (BIA-core 3000 Instrument Handbook). The analyte - ligand complex was allowed to associate and dissociate for 3 and 5 min, respectively, with background subtraction using a flow cell that was subjected to the coupling reaction but without protein, as reference surface. For concentration series, Pep19-2.5 was tested at 0.5, 1, 2, and 3 µM. For inhibition experiments LPS (1 µM) was preincubated with 3 µM Pep19-2.5 and the mix was injected over the HK surface. The surface was regenerated with a 15 s injection of 50 mM NaOH and 5 min buffer flow at the end of each binding cycle. The data from the BIAcore sensorgrams were fitted locally, using the one-step biomolecular association reaction model (1:1 Langmuir binding).

### Preparation and treatment of PBMCs

PBMCs were isolated using diluted buffy coat (1:1 in PBS) from healthy volunteers as described^19^. The PBMC cell layer was collected, cells were washed twice in PBS and resuspended in RPMI medium (Invitrogen). PBMCs (1 × 10^6^ cells/ml) were treated with combinations of LPS (10 ng/ml), Pep19-2.5 (100 ng/ml), HK (1 µg/ml), or medium alone (final volume of 1000 µl). After 4 h or an overnight incubation (20 h) on rotation at 37 °C, cells were centrifuged (400 x g for 20 min). Supernatants were kept frozen (−80 °C) until use. The cell pellet was resuspended and used for FACS analysis or clotting assays.

### FACS analysis

PBMCs (1 × 10^6^ cells/ml) were treated with combinations of LPS (10 ng/ml), Pep19-2.5 (100 ng/ml), or medium alone (final volume of 1000 µl), for 4 h at 37 °C on a rotator. Cells were then washed in PBS, and incubated with FITC– anti-TF IgG (Lifespan Biosciences), a FITC-conjugated isotype control antibody (Immunotools), or PE–anti-CD14 IgG (Biolegend) for 30 min on ice. Samples were washed and analyzed in a BD Accuri C6 flow cytometer (Becton Dickinson). Monocytes were identified by forward/ side scatter characteristics and CD14 expression^33^.

### Cytokine ELISA

The concentrations of IL-6, TNF-alpha and IL10 in the supernatants from PBMCs were deter- mined by ELISA according to the manufacturer’s protocol (R&D Systems).
